# Getting to “Yes”: Overcoming Client Reluctance to Engage in Chair Work

**DOI:** 10.3389/fpsyg.2020.582856

**Published:** 2020-10-06

**Authors:** Peter Muntigl, Adam O. Horvath, Lynda Chubak, Lynne Angus

**Affiliations:** ^1^Faculty of Education, Simon Fraser University, Burnaby, BC, Canada; ^2^Department of Anthropology, University of Toronto, Toronto, ON, Canada; ^3^Department of Psychology, York University, Toronto, ON, Canada

**Keywords:** affiliation, chair work, conversation analysis, directives, deontics, emotion-focused therapy, recruitment

## Abstract

**Goals:**

Securing clients’ active and enthusiastic collaboration to participate in activities therapists would like to implement in therapy (e.g., free association, *in vivo* exposure, or the engagement in chair work) is a core mission in therapy. However, from the clients’ perspective, these tasks frequently represent novel challenges that can trigger anxiety and reluctance. Thus, a key element in therapy is the negotiation between therapist and client to move beyond such reluctance to potentially effective therapy activities and, at the same time, maintain positive relational affiliation between therapist and client. In this research we examined (1) a collection of therapist proposal/client response sequences that were geared toward recruiting participation in *chair work* and (2) sequences containing hesitation or instances where decisions to engage in chair work were deferred and related relational disaffiliation. Our goal was to identify the conversational resources (both verbal and non-verbal) that worked to reject a proposed activity (or convey impending rejection) and examine the interactional practices directed at resolving client reluctance.

**Method:**

We used the conceptual and methodological resources of Conversation Analysis to examine a corpus of proposal/response sequences that targeted chair work entry in Emotion-focused Therapy.

**Results:**

The resulting data set included some smooth and successful engagements and others more challenging, involving clients delaying or resisting engagement with chair work. Clients were found to defer or refuse engagement through a range of resources such as withholding a response (silence), questioning the authenticity of the task, or directly refusing. We identified specific therapist practices that facilitated engagement in “refusal-implicative” contexts such as proffering “or” alternatives, offering extended rationales for the activity (accounting), and elaborating on the proposals. We observed that the therapists’ deontic stance (mitigated and reduced claims to authority) and moderated epistemic positioning (deference to the client’s primacy of knowledge and information) played an important role in facilitating engagement.

**Conclusion:**

Our research highlights the kinds of interactional sequences in which clients and therapists are able to achieve alignment in mutually working toward chair work entry. Based on these observations, we offer some practical advice to therapists in formulating proposals to engage clients during in-therapy work.

## Introduction

There is an accumulation of evidence that therapists and clients who can agree on the importance of the in-therapy activity proposed by the therapist, and actively collaborate in these tasks, have more successful outcomes than those who struggle to achieve such consensus (Hatcher et al., 1995; Del Re et al., 2012). These findings are consistent with clinical wisdom and are closely mirrored by Bordin’s (1975, 1979, 1994) hypothesis that, across different modes of psychotherapy, the alliance in general and the task component of the alliance in particular (i.e., prizing of, and engagement in, the therapist proposed in-therapy tasks) is the core feature of the productive therapy process (Bordin, 1975; Horvath, 2018; Flückiger et al., 2020). However, while the relation between the task component of the alliance and outcome is well documented (Horvath and Bedi, 2002; Flückiger et al., 2018), much less is known about the process of how such consensus is interactively achieved and how clients’ reluctance to engage in proposed therapeutic activities is resolved in clinical practice.

Our research program was designed to make inroads toward the better understanding of these processes by closely examining clinical examples of sequences involving specific task negotiations. To explicate these processes, we are utilizing the conceptual and methodological resources of Conversation Analysis (CA) (Heritage, 2004; Peräkylä et al., 2008; Sidnell and Stivers, 2013) that allows us to focus on the communicative sequences that participants use to achieve consensus with respect to therapists’ proposals to engage in in-session tasks. In contrast to more traditional lenses used in psychotherapy research that tend to focus on intent and cognition (i.e., the mental process that motivates therapist and client to do or to resist such activity), the CA approach compliments this perspective by prioritizing observable social conduct: How agreement is achieved in conversation; what kinds of interactive resources (verbal, prosodic, and non-verbal) were put in play and in what kinds of sequences? We focus on the interactional ways participants indicate compliance or reticence, communicate lack of affiliation, and so on. We also draw from prior CA research on conversational directives and *deontics* (Couper-Kuhlen, 2014; Stevanovic and Peräkylä, 2014; Stevanovic and Svennevig, 2015), to better understand how increasing degrees of difficulties to task consensus are realized in therapy dialog and how the kinds of sequences that result in more or less successful resolution unfold.

For the current study we chose to examine clinical examples of therapists’ and clients’ negotiations to engage in a specific therapeutic task: chair work (Greenberg, 1979). Chair work (including both the “empty-chair and the “two-chair” variety) involves the client re-engaging with an unresolved, problem-laden interpersonal situation in a kind of role-play, giving voice, in turn, to different aspects of the unresolved conflict (“split”): The “empty-chair” variant most often involves a relationship with a significant other person, while the two-chair version most often focuses on two (or more) dis-owned aspects within the client, commonly referred to as “splits.”^1^ In all instances, the goal is to bring the unresolved/split dynamics into the present, here-and-now, of the therapy session, and to help the client move toward resolution or accommodation of the conflictual elements (Greenberg and Higgins, 1980). Because chair work is an expressive, here-and-now enactment that uses imagery and active expression, it is often accompanied by the activation and intensification of painful emotions. For this reason, hesitation, performance anxiety, shame, and/or awkwardness may be associated with this task. As a consequence, some clients may not feel “ready” – or *are reluctant to engage the task* –to participate in what can be an unfamiliar and emotionally intensifying experience.

Chair work is a frequently used and well researched intervention which, at the early stages when the therapist invites the client to engage in it, shares many of the same challenges to gaining task compliance irrespective of the treatment modality or specifics of client issues. Accomplishing “consensus-based” decisions is a common aim in many mental health care contexts (Valkeapää et al., 2020). Achieving task-consensus to do chair work requires that therapists and clients come to parallel orientation to the actual task, and, at the same time, align or realign themselves relationally while confronting this novel task that likely generates a degree of anxiety and tension for the client. This later duality gives us an opportunity to explore the negotiating process from both the instrumental and relational perspectives.

## Directives in Social Interaction

Getting others to do things is a pervasive activity in social interaction. These action types, commonly referred to as *directives*, involve some future event or task to be accomplished, orient to speakers’ rights and responsibilities, and make relevant some form of acceptance or compliance by the recipient or commitment to carry out the task (Couper-Kuhlen, 2014). Various additional pragmatic dimensions are important to consider when examining directive environments, especially involving imperative formats, such as participant role distributions (participation frameworks), the relation to the ongoing activity and the degree of immediacy or urgency (Sorjonen et al., 2017). Directives may include a variety of action types such as requests, commands, proposals, or suggestions (Couper-Kuhlen, 2014; Landmark et al., 2015; Stevanovic and Svennevig, 2015). The ways in which directives are formulated (e.g., the expressions, words used to design the directive) tend to orient to certain kinds of general principles that involve *entitlement* and *contingency* (Drew and Couper-Kuhlen, 2014). For example, the degree of entitlement to direct another’s actions (e.g., assigning homework; giving advice concerning a problem) is often realized in the linguistic design of the directive, such as whether imperative or declarative formats or whether certain modality markers (e.g., will, would, could, should, etc.) are used (Heinemann, 2006; Craven and Potter, 2010). These displayed sensitivities to the speakers’ role relationships have also been shown to take account of the participants’ agency with regard to who is being mobilized to act, including who will potentially benefit from the future action, if carried out (Clayman and Heritage, 2014; Drew and Couper-Kuhlen, 2014). There may also be various reasons for which a recipient may refrain from complying with the directive. Thus, a speaker can orient to these contingencies by making the directive less likely to be refused. For example, prefacing a directive with “I wonder” displays that the recipient may have other (perhaps better) options (Curl and Drew, 2008), and this sensitivity to the other’s concerns can make it easier for the recipient to accept the terms of the directive. How speakers design their directives will also be predicated on what Rossi (2012) has termed “low cost” vs. “high cost” actions. Thus, therapists will presumably not need to do much discursive work in getting their clients to take a seat, but for higher cost actions, such as getting clients to engage in chair work, presumably more work will need to be done.

More recently in CA work, this broad spectrum of actions that involves directives (but also *commissives*, such as offers and invitations) has been examined under the general rubric of *deontics*, and more specifically *deontic stance* and *deontic status* (Stevanovic and Peräkylä, 2014). According to Stevanovic and Svennevig (2015:2), “Deontic stance refers to the participants’ public ways of displaying how authoritative or powerful they are in certain domains of action relative to their co-participants, and deontic status denotes the relative position of authority and power that a participant is considered to have or not to have, irrespective of what he or she publicly claims.” Further, entitlement and contingency hold a central place within this framework for understanding how these kinds of (authoritative) role relationships are negotiated turn by turn.

Directive sequences are commonly found in therapeutic approaches. For example, in chair work, a technique that is regularly used in Emotion-focused and Gestalt therapies, therapists need to recruit clients into this activity, *in situ* (Sutherland et al., 2014; Muntigl et al., 2017). In Cognitive Behavioral Therapy (CBT), therapists often make proposals to clients for homework or future behavioral change (Ekberg and LeCouteur, 2015).

## Opposing Directives

The sequential management of disaffiliation, or of episodes in which disagreement or the withholding of agreement occurs, is a burgeoning topic in CA-focused psychotherapy research. These studies have been examining the sequential environments of questioning and formulating/interpreting (MacMartin, 2008; Vehviläinen, 2008; Voutilainen et al., 2011; Muntigl, 2013; Muntigl et al., 2013; Weiste, 2015). CA research on directive sequences (i.e., invitations, offers, requests and proposals) has been investigating the kinds of interactional features that may be signaling rejection and, moreover, how speakers orient to this form of interactional challenge. For example, it has been shown that silence following an initiating directive action in everyday contexts generally implies potential rejection, and that speakers frequently produce a *subsequent version* of the directive, with the aim of gaining eventual compliance (Davidson, 1984). When rejections are more overtly expressed, it has been found that they are often accompanied by accounts (Heritage, 1984) or even, in the case of invitations, that an account may be offered in place of the rejection (Drew, 1984). There is an extensive literature and history of accounts and accounting practices in CA (see Levinson, 1983; Heritage, 1984; Antaki, 1994). In general, accounts perform some kind of “explanatory” work. However, in CA research, the function associated with the account will always be examined with respect to its place and organization within a sequence (Buttny, 1993; Antaki, 1994). Accounts have been shown to appear in a variety of sequential locations and, most notably, in dispreferred responses in which an explanation is given as to why the “preferred” response (e.g., acceptance) will not be given. Buttny (1993:62) points out that accounting is an interactional achievement and “how they [accounts] are ordered and produced is contingent in part on the recipient”. This can be taken to mean that the place in which accounts may appear is shaped by a recipient and may arise where some form of interactional trouble is looming. Accounts have been shown to regularly occur in advice giving sequences in Cognitive Behavioral Therapy. Ekberg and LeCouteur (2015) have found that clients tend to cast their rejections as an inability to comply with the therapist’s future proposals. They identified three different types of accounting practices following therapist offers of advice (e.g., how to better manage a daughter’s behavior; proposing alternative ways to change own behavior; going for a walk after work, rather than drinking alcohol): appeals to restrictive situational factors (e.g., inability, not having the resources); appeals to a fixed physical state (e.g., being too tired); and assertions of previous effort to do what the therapist was proposing (e.g., the client had already tried it).

Watson and Greenberg (2000: 181) claim that clients in Emotion-focused Therapy are often hesitant to engage in task-related activities such as chair work for a variety of reasons. They may be overly cautious when asked to experience their feelings, they may be scared of losing control, and they may find the proposed task awkward and artificial or not to be relevant. These reasons for the clients’ refusals of task-based activities are mainly taken from an “intrapersonal” perspective, what clients feel in certain situations. Our CA approach compliments this perspective by focusing on how opposition (whatever the etiology) is displayed publicly and interactively negotiated. We build on these findings gleaned from the application of CA research on discourses involving directives in general by here focusing particular attention to the relation between the therapist’s deontic stance and epistemic positioning and the degree of success or opposition in negotiating chair work.

## Data

The overarching goal in this study is to identify and analyze the interactional resources used in therapy to achieve cooperative engagement with respect to challenging in-therapy tasks. In clinical practice there are a great variety of tasks that therapists may wish to get clients to do, for example: *in vivo* practices, rehearsals of physical behaviors (e.g., relaxing exercises), free association, etc. Each of these tasks has unique features that influence the structure of the interaction. In order to focus on the generic aspects of the process—how these negotiations are realized—we chose to focus on a single specific task and context: negotiating participation in chair work (CW) within the framework of Emotion-focused/Process Experiential Therapy (Greenberg et al., 1993; Greenberg, 2004). Our data is drawn from the York I Depression Study (Greenberg and Watson, 1998).^2^ Eight cases involving video and audio-recordings of clinically depressed clients undergoing emotion-focused treatment (6 females, 2 males) were made available to us. Four of the cases involved recovered clients and 4 were non-recovered. Cases were selected based on the following criteria: completeness of recordings (all sessions taped) and quality of recordings (i.e., best quality visual and audio).^3^ The 5 participating therapists (all female) were experienced, trained and supervised in Emotion-focused Therapy/Process-Experiential treatment. For each case, we were supplied with three 1-h long videotaped psychotherapy sessions—from the beginning, middle and late phases of therapy—bringing our total number of sessions in our data to 21. Sessions from each phase were selected according to quality of recording and completeness (both audio and video recordings) rather than session number. Written informed consent was obtained from the participants for the publication of anonymized data. Persons referred to within therapy, including the client, have been given pseudonyms.

## Methods

In this project, we used the methods and conceptual framework of CA (Sidnell and Stivers, 2013), taking into account the standards for qualitative research as outlined in Levitt et al. (2018). Generally, CA aims to identify and describe recurring practices of social interaction (Sidnell, 2013), in which speakers are found to organize their turns at talking and their unfolding sequences of actions such as, for example, answers following questions and compliance following requests (Heritage, 2004). Much analytical energy is often used to illustrate the, sometimes subtle but highly relevant, variations within a practice or sequence, showing how a question or request may be designed in different ways (often having different implications for next response) and how a “recipient” of a first action has different choices of responding (Sidnell, 2013). CA analytic claims are made to abide by standards of *transparency* and *validity* (Peräkylä, 2004), which bear similarity to what has been called *trustworthiness*, a term that is used in other qualitative approaches (Levitt et al., 2018). Transcripts of talk of which analytic claims are based are published alongside the analysis, thus leaving the claims open to inspection and challenge by readers. Validity is gauged with respect to the ‘next turn proof procedure’, which argues that speakers display their understanding of a prior utterance and that the analyst’s interpretation should aim to reflect that understanding (Sacks et al., 1974), and not deviate from it, for example, by offering a more abstract interpretation. Although CA studies often draw from a variety of “cases” (i.e., different sets of participants), the analytic focus is placed primarily on the recurring practice itself, irrespective of who specifically is participating in the social interaction. This is not taken to mean that the participants involved are not important or unique. Rather, the analysis seeks to draw attention to how certain interactional goals are regularly accomplished and the various trajectories used to (or fail to) get there.

### Corpus Selection and Transcription

Authors 1 and 3 examined each of the sessions for the occurrence of chair work and found that 18 sessions contained this task-based activity. Although therapists and clients were found to commonly exit and then somewhat later re-enter chair work, we have restricted our focus to first-time entry within a session. These eighteen sessions containing an instance in which the therapist proposed chair work were then selected for further analysis. Each session was transcribed according to conversation analysis (CA) transcription conventions outlined in Jefferson (2004), and further guided by Hepburn and Bolden (2017) and Mondada (2019). Author 3 did all initial transcription work. Author 1 later re-visited all chair work segments and modified the transcripts where appropriate. For space and readability, extracts presented in this paper have been abridged and are slightly simplified versions of the original transcripts. The transcription conventions used in this paper are shown in Table 1.

**TABLE 1:** Transcription notation.

**Symbol**	**Meaning**	**Symbol**	**Meaning**
[	Starting point of overlapping talk	↓word	Markedly downward shift in pitch
]	Endpoint of overlapping talk	↑word	Markedly upward shift in pitch
(1.5)	Silence measured in seconds	.hhh	Audible inhalation, # of h’s indicate length
(.)	Silence less than 0.2-s		
.	Falling intonation at end of utterance	hhh	Audible exhalation, # of h’s indicate length
,	Continuing intonation at end of utterance	heh/huh/hah/hih	Laugh particles
?	Rising intonation at end of utterance	wo(h)rd	Laugh particle/outbreath inserted within a word
(word)	Transcriber’s guess		
()	Inaudible section	hx	Sigh
wor-	Truncated, cut-off speech	∼word∼	Tremulous/wobbly voice through text
wo:rd	Prolongation of sound	.snih	Sniff
word = word	Latching (no audible break between words)	huhh.hhihHuyuh	Sobbing
<*word*>	Stretch of talk slower, drawn out	>*hhuh*<	Sobbing—if sharply inhaled or exhaled
>*word*<	Stretch of talk rushed, compressed	((cough))	Audible non-speech sounds
°word°	Stretch of talk spoken quietly	*italics (blue)*	Non-verbal behavior (actor indicated by initial)
word	Emphasis		
WORD	Markedly loud		

### Identifying Directive Sequences

Prior research has found that entering chair work in Emotion-focused Therapy (EFT) is regularly accomplished through four distinct interlocking interactional phases: (1) formulating the client’s trouble; (2) recruiting participation in chair work; (3) readjusting the participation frame; (4) making contact (Muntigl et al., 2017). This current paper expands upon this earlier analysis by delving deeper into Phase 2 and, more specifically, by focussing on directive sequences in which therapists seek client agreement on subsequent engagement in chair work.^4^ We trace how the therapist interactionally manages to engage the client’s participation, especially in those contexts in which client agreement to engage in chair work is not immediately forthcoming or even contested. The method of identifying and selecting a corpus of directive sequences is taken from Muntigl et al. (2017). These sequences begin with a therapist’s directive action and are completed when client ratification or refusal occurs. Ten sessions from 4 cases of this previous investigation were included in this study and 8 more sessions from 3 additional cases were then added (those containing chair work), applying the same method of identifying chair work phases and directive sequences. Author 1 did the initial sequence analysis and identification of proposal sequences into types. Authors 2 and 3 later re-visited the analysis by checking for appropriateness of sequence-type identification and by inspecting (and elaborating on) the turn-by-turn analysis.

## Proposal Sequences in Chair Work

From the directive sequences analyzed, it was found that most initiating directive actions functioned as *proposals*. These are actions that invite the recipient’s involvement as opposed to presupposing or demanding it (Stivers and Sidnell, 2016) and position the recipient as both the agent and beneficiary of the action to be carried out (Clayman and Heritage, 2014; Couper-Kuhlen, 2014). With few exceptions, proposals were designed in a highly contingent manner (e.g., involving pre-, in-turn hesitation, heightened/softened pitch, deontic modality of “willingness” or possibility/choice, rising intonation, head tilting), orienting to the client’s greater entitlement to decide over the suggested course of action. Turn features commonly referenced the shared nature of the task: “*we* could/should”, “*we* can”, “can *we*”), and client willingness: “would you be willing”, “you need”). Proposals often included a deictic *that*/*this*, indexing a shared understanding of what “work” is being done.

Chair work involves the recall and re-experiencing of, in the present, issues that the client has had difficulties with.^5^ As such, by its very nature, it is potentially stress inducing and the client may be reluctant to consent to engage. The degree of ensuing reticence or opposition poses different levels of challenges and requires different interactive resources to resolve or overcome. To explicate the relation between the degrees of client opposition/reluctance and the kinds of conversational resources used by therapists we subdivided the available examples from our database into three broad categories: *Smooth Entry*; *Mediated Entry*; and *Opposition to Entry*. Smooth entry (*n* = 5) involves sequences in which the therapist’s proposal to do chair work around a specific conflict/emotion is followed by the client immediately endorsing the suggested project. Mediated entry (*n* = 15) is marked by delays in clients providing a response, prompting the therapist to do more interactional work to pursue eventual engagement in chair work. Opposition to entry sequences (*n* = 6) include client actions that challenge the value or validity of the intervention or that directly refuse participation in the activity. Although more than two-thirds of proposal attempts led to eventual engagement in chair work, in some cases chair work was abandoned following the client’s opposition.

### Smooth Entry: Proposal Sequences With Affiliative Uptake

In the smooth entry examples, there was only one attempted turn at proposing chair work before the client agreed. Proposals were designed in a highly contingent manner (e.g., involving hesitation, heightened pitch, deontic modality of “willingness”, rising intonation, head tilting), orienting to the client’s greater entitlement to decide over the suggested course of action.

• **w- wudja** be **willing** to do it**?**
• **↑**d’yu **wannu** uhm
• >so is that< something that you’**d like**^∗^to try tuh (.) do:**?**
• **↑wanna** work with that today**?**
• **.h** >so is that< something that you’d **like to try** tuh (.) do:**?** then is.hh **at least try** to **(0.8)** work toward:

Additionally, in smooth entry, therapist proposal turns commonly feature either a deitic “that” only or “that conflict” as something to work on, displaying that the therapist and client have come to a clear, shared understanding of the in-the-moment conflict. This feature can also frequently be found in turns proceeding agreement, and after previous more extensive turns have been made in scenarios with dissent or rejection.

• so we can work with **that** some more today?
• >so is **that**< something that you’d like ^∗^to try tuh (.) do:?
• ↑wanna work with **that** today?
• .hhh is **that conflict** something that (.) we should (.) spend some time on?”

A sequence of smooth entry with the client Owen, who is a student that is also working part-time, is seen in Extract 1.


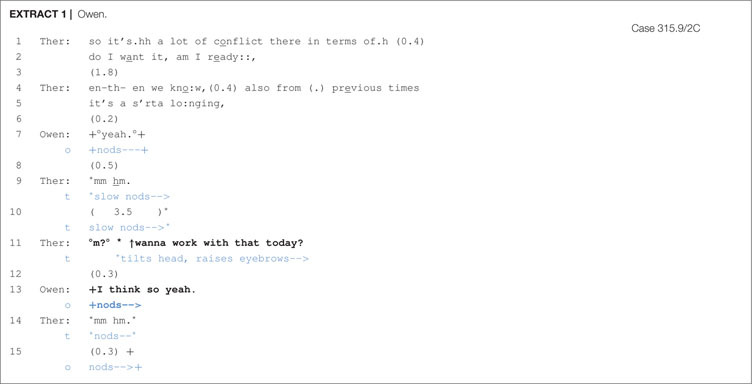


In lines 1–5, the therapist orients to Owen’s dilemma by reformulating Owen’s description of his desire for and uncertainty about wanting a relationship as a source of conflict and connecting it to previous discussions as “a s′rta lo:nging,”. Following this, Owen affiliates with this formulation through verbal acknowledgement and nodding (line 7), leading the therapist to first confirm Owen’s acknowledgement and then, in line 11, to produce a proposal: “°m?°↑wanna work with that today?”. By targeting the client’s ‘willingness’ (i.e., wanna), the therapist orients to the client’s greater entitlement to decide over the suggested course of action. What is implied through this turn format is that Owen will not only play an agentive role in the impending action, but will also be a beneficiary of the action; that is, what is being suggested will have therapeutic gains for the client. Her granting Owen the prerogative to proceed or not is also designed in a highly contingent manner, involving hesitation, heightened pitch, rising intonation, and head tilting. Owen’s verbal response of “I think so yeah.”, while nodding (line 13), occurs smoothly and quickly and endorses the therapist’s proposal for chair work.

Beginning chair work with a different client, Lisa, is shown in Extract 2 and illustrates how a therapist adds more specificity to their proposal, indicating right from the start whom the chair work will target and that the activity will be beneficial.


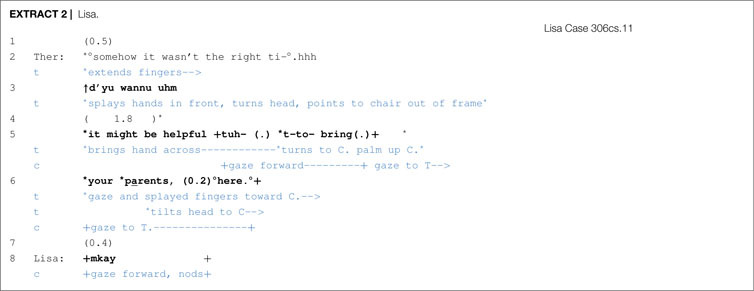


In line 3, the therapist begins to put out her proposal while pointing to the empty chair, which provides some clarity regarding what the therapist will be directing the client to do. Then, in line 5, the therapist continues by mentioning the value (helpful) and the aim (t-to- bring (.) your parents, (0.2) °here.°) of chair work. Contingency is displayed via mitigation (might be) within turn pausing, a proffering gesture with the hand and head tilting. Agreement/compliance occurs in line 8, both vocally and non-vocally. Lisa’s response is pro-social, thus affiliative, and endorses or aligns with the activity in progress (leading up to chair work).

### Mediated Entry: Therapist Practices for Pursuing Engagement

For most proposal sequences examined, client compliance was not immediate, but deferred. This delay in responding was often signaled by pauses (silence) on the part of the client and by non-vocal actions that could be interpreted as a form of disengagement with the therapist’s suggested course of action. In contexts of silence following a proposal, we found that therapists would pursue compliance, not by immediately offering another version of the proposal (cf. Davidson, 1984), but through a variety of interactional practices that highlighted the contingencies associated with making the proposal and the client’s upgraded entitlements in deciding the future course of action. In some of the cases, we observed that therapists would put direct pressure on clients to respond (and by implication accept), whereas other practices worked in a more subtle fashion by adding more background or relevant circumstantial information to the proposal, making the rationale behind the proposal more transparent. Four practices were identified: (1) Offering an “or” alternative (*n* = 2); (2) Providing an account (*n* = 6); (3) Elaborating on the conditions for proposing the activity (*n* = 4); and (4) Requesting confirmation (*n* = 3).

#### “Or” Alternative

When confronted with a delay following a proposal, therapists had the option of appending an *Or*-prefaced alternative to their turn. This practice is seen in Extract 3, during which the therapist attempts to engage the client Jennifer in chair work.


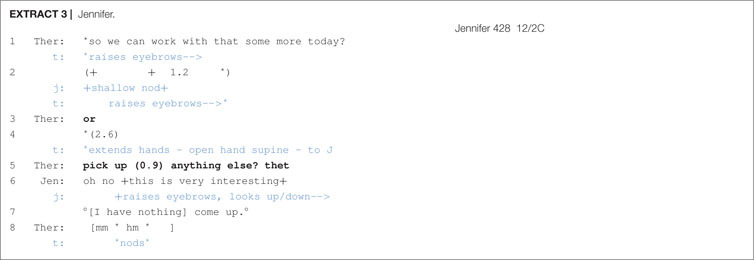


Following the therapist’s proposal (line 1), there is a significant 1.2 s pause during which Jennifer gives a shallow nod. The therapist then, in line 3, appends an *or* onto her prior turn, which functions in a couple of ways. First, it treats Jennifer’s nod as insufficiently displaying acceptance and, second, it gives Jennifer an opportunity to suggest an alternative course of action–thus obviating the need for her to refuse the therapist’s proposal if need be–and downgrades the force of the proposal. Further, the extension of the therapist’s hands, as an open hand supine (OHS) gesture, toward the client may be seen as an offer (Kendon, 2004) and works to reinforce the downgraded deontic stance set in motion by the stand-alone *or*. Following no response from the client, the therapist continues her turn by supplying an alternative course of action (line 5) and this then immediately receives acceptance by Jennifer of the therapist’s original proposal of line 1.

In a study of polar question sequences, Drake (2015) found that turn-final *or* in these sequential environments would index a downgraded epistemic stance or “a lack of commitment to the expressed proposition” (p. 305). This is because this kind of turn format torques preference structure in favor of disconfirmation and opens the floor to possible alternatives. For proposal sequences, however, the orientation is not toward propositions or epistemics, but rather to deontics and the ‘orchestration of action’ in terms of offers, directives, requests, etc. But otherwise, the function appears to be similar. *Or* in these sequential environments, as shown in Extract 3, may be seen to index a downgraded deontic stance in which the client’s obligation to comply is mitigated. Space is given to clients to consider alternative responses and, further, an opportunity to refuse (respond with a dispreferred alternative) is created. Additionally, the *Or*-prefaced alternative may ease up the pressure of complying, making it less difficult for clients, such as Jennifer, to deflect the challenging task of chair work.

#### Accounting

Another therapist practice dealing with delays in responding is *accounting*. These were found to come in two basic formats: Providing an explanation for how the proposal may benefit the client; Providing a justification that highlights the importance of doing chair work. In Extract 4, the therapist is attempting to get Sofia to speak with her father and her turn orients to contingency and, following no response, provides an account that explains how engaging in chair work may help her to work through her pain.


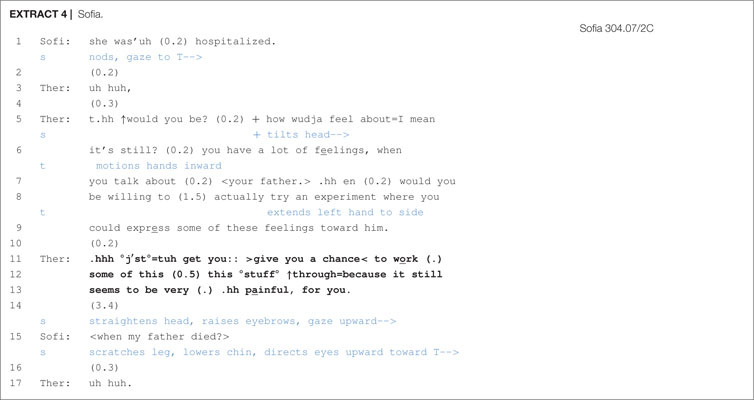


The therapist’s proposal in lines 5 to 9 is packaged with many features of contingency: a beginning proposal that is self-repaired (line 5), formulating the activity as an *experiment*, modality of ability (*could* express), willingness (would you be willing to) and non-vocal actions (head tilt, extending left hand to side). Following a brief pause in line 10, which signals potential rejection, the therapist continues with an account that indicates the benefits (give you a chance< to work…) and rationale (still seems to be very (.).hh painful, for you) of chair work. There is a significant delay in Sofia’s response (line 14) and she also seems to bodily disengage from the therapist by gazing upward and away from the therapist. She then initiates an other-repair, requesting the therapist to clarify the details surrounding her proposal. It would appear that there remains some doubt regarding which painful feelings in relation to her father the therapist is referring to, leading Sofia to this other-repair request in which she seeks confirmation as to whether it was the event pertaining to her father’s death. After the repair sequence is resolved (not shown in the extract), Sofia does eventually concede to the proposal.

An account that provides a justification for the importance of doing chair work is illustrated in Extract 5 with the client Jennifer.


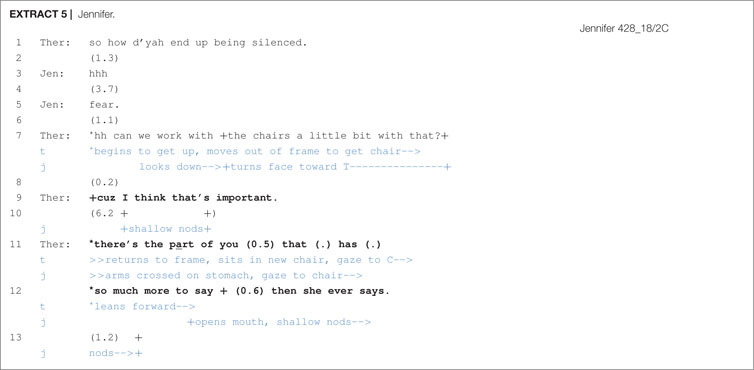


During the therapist’s proposal in line 7, Jennifer only partially bodily engages with the therapist by looking down and turning her face toward her. After a brief pause, the therapist provides a justification by first mentioning the significance of doing the activity (I think that’s important) and then proceeds to indicate why it would be important to do. During this time, the client expresses token affiliation through repeated nods and, toward the end of the account, the therapist draws closer to the client, decreasing the physical space between them. Because proposals for chair work involve a fair degree of emotional commitment and involvement from the client, it may not always be easy for clients to readily comply. Thus, in contexts where clients appear hesitant or reluctant, further explanation and justification may be helpful in reassuring clients of the potential value or benefit of doing this activity.

#### Elaborating

In the following excerpt we illustrate the practice of elaboration by the therapist. In entering chair work, there are many details to work out. These range from setting up a new spatial arrangement to perform the task to launching a new participation framework in which the actor roles are to be established. Thus, another way to pursue acceptance to the proposal is to elaborate on these conditions by adding more specificity to what is going to happen. This is illustrated in Extract 6 with the client Ernie.


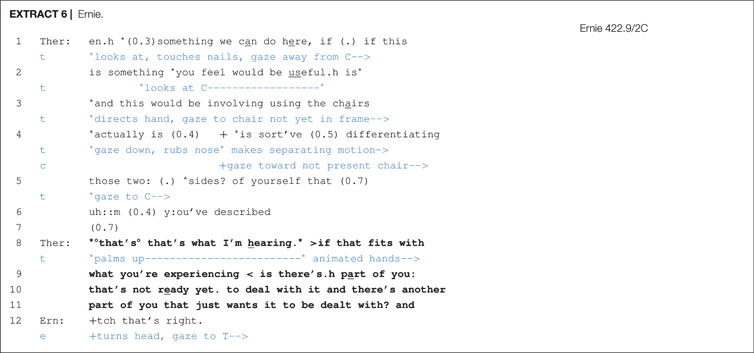


The therapist is proposing two-chair work in which Ernie begins a dialog by acting out two different sides of himself. There are numerous expressions indexing contingency (can do; you feel would be useful; actually; sort’ve (0.5) differentiating) and thus an orientation to the client’s greater entitlement to ratify the activity. Toward the end of the proposal, lines 5–6, the therapist pauses at points where the client could offer some form of non-vocal token affiliation (by nodding, for example) concerning the two sides that he had just previously described, but does not. Further, although the therapist references these two sides, he does not specify what they are. A silence occurs in line 7 that may imply an impending rejection, but the therapist grabs another turn-taking opportunity in order to elaborate on the two sides. First, the therapist adopts a downgraded deontic stance through evidential markers (that’s what I’m hearing) and by checking her understanding (if that fits with what you’re experiencing), thus allowing the client to take up upgraded epistemic and deontic rights. Next, the therapist adds more *granularity* to her prior description (Schegloff, 2000) by adding more specificity to what these two positions consist of (‘not ready yet’ vs. ‘just wants it to be dealt with’). The therapist also orients to contingency non-vocally in line 8 by animating her hands in a palm up position, suggesting that what she is proposing is a possibility and that the final decision (deontic authority) will rest with Ernie. The client then voices his confirmation of his inner conflict in line 12.

This practice of elaborating and of making descriptions more granular is, we would argue, being done in the service of securing client affiliation. Just as with Stivers’ (2008) observations on story telling, in which tellers make their descriptions more granular to provide recipients with more access to the event in question, thus allowing the recipient an opportunity to affiliate with the telling, so do therapists make their proposals more granular to better specify the conditions surrounding the proposal and help clients to better understand what will be required of them.^6^

#### Seeking Confirmation

The fourth way of dealing with delays following a proposal was the therapist practice of seeking confirmation after the delay. Confirmation seeking appears in various turn formats such as “that’s o↑kay?”, “is that alright?”, “what you think about that.”, “yeah?”. Confirmation seeking is shown in Extracts 7 and 8.


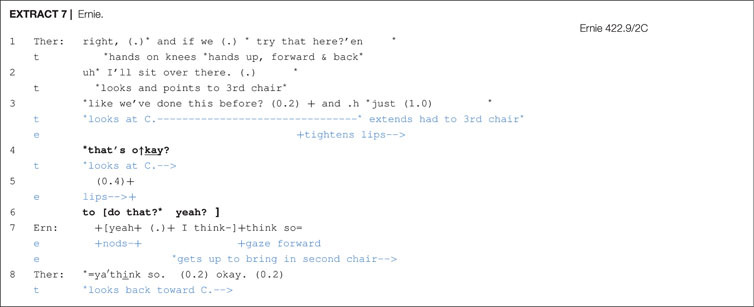



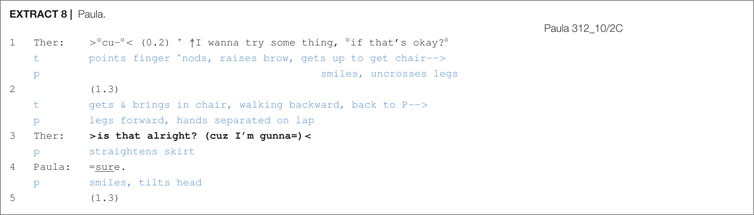


In Extract 7, following a 1 s silence in line 3, the therapist seeks confirmation with turn-final rising intonation “that’s o↑kay?”, which is followed by a brief pause without any client acknowledgement and, in line 6, two more confirmation seeking expressions (to do that?; yeah?). Ernie, in line 7, produces overlapping acceptance. In Extract 8, a 1.3 s silence follows the proposal. Then, as in Extract 7, the therapist seeks confirmation with turn-final rising intonation (>is that alright?), which receives immediate confirmation from the client Paula.

According to Stivers and Rossano (2010:27), directive actions such as requests and offers are “high in response relevance”, meaning that they strongly mobilize a response from the recipient. Further resources that consist of lexico-grammar, prosody, gaze and epistemic domain (i.e., recipient’s degree of/access to knowledge) play a crucial part in strengthening or weakening response relevance. Clients nonetheless sometimes delay their response and one therapist technique for increasing response relevance, as shown in Extracts 7 and 8, is to append a confirmation seeking tag after a prolonged silence. This puts further pressure on clients to respond and, as these extracts illustrate, it is met with success.

### Opposition: Managing Client Explicit Refusals

A step up from delaying the response to a proposal, and thus expressing impending rejection, is to explicitly *do* a refusal. Two practices of refusing were found in the data: Questioning the authenticity of the activity–refusal by implication–and direct refusals.

#### Questioning the Authenticity of the Activity

Participating in chair work requires that clients are ready to experience and engage with their emotions in the presence of an imagined other or conflicted self. According to Watson and Greenberg (2000: 181), “they [clients] may find the activities required of certain tasks too artificial and contrived, and feel silly performing them, for example, when asked to talk to an empty chair.” Extract 9 shows a client’s difficulty in accepting and going along with the ‘imaginative’ aspect of chair work.


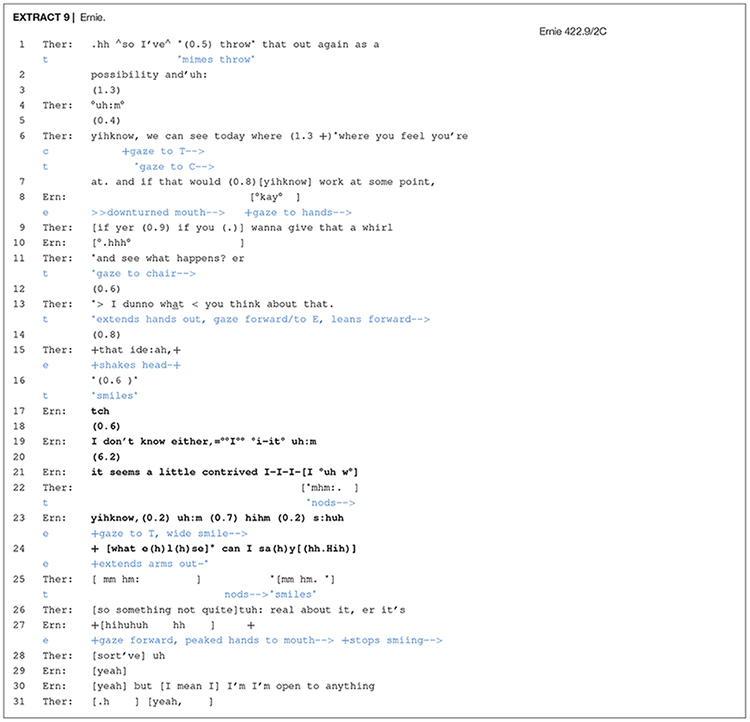


In this instance of pre-chair work, the therapist is proposing that Ernie express his emotions to his imagined-in-the-moment ex-wife. The therapist’s proposal contains a number of turn design features that cast it as unmotivated and conditional on the client’s interest: “throw that out again as a possibility” (lines 1–2); “if that would yihknow work at some point” (line 7). The many pauses, the term “possibility”, the expression “where you feel you’re at” and conditional *if* all work together in constructing this proposal as highly contingent on the client’s acceptance, but also display a moment-by-moment orientation to his lack of affiliative displays. For instance, Ernie does not only refrain from accepting at numerous places where he could have, but he also delivers muted agreement that is accompanied by non-vocal actions that signal displeasure and disengagement (line 8) and a long whispered in-breath possibly displaying distress (line 10). In response, the therapist immediately follows up with one more attempt in which she orients to Ernie’s potential willingness (“wanna give that a whirl”). Following no response from Ernie, she then solicits an assessment (and confirmation) from him pertaining to her suggestion to do chair work (“> I dunno what< you think about that.”) and, later in line 15, provides more granularity (Schegloff, 2000) to deictic *that* by elaborating with “that ide:ah”.

At this point, Ernie’s refusal becomes more overt. He shakes his head in line 15 and then, following a few pauses and a “tch”, he grammatically ties his turn to the therapist’s (line 13) by offering a parallel claim of uncertainty about engaging in chair work (“I don’t know either”). In line 21, Ernie makes his discomfort explicit by pointing out the artificiality of the proposed task: “it seems a little contrived I-I-I-I °uh w° yihknow,”. In lines 22 and 25, the therapist displays affiliation with the client’s initial reluctance by a number of acknowledgement tokens (mm hm), nodding, smiling and then by producing a formulation that endorses Ernie’s unease concerning the artificial quality of the proposed activity (“so something not quite uh: real about it”). These therapist actions, which work to re-affiliate with the client’s opposing viewpoint, engender a movement toward realignment with the activity (see also Muntigl et al., 2012; Muntigl et al., 2013). The realignment is successful, and Ernie voices his willingness to comply (lines 29–30). This extract not only illustrates the level of attention required to track subtle non-vocal indicators (a shift in gaze, pausing), but also how the therapist responds to this by downgrading her epistemic position responsively (what< you think about that.). This shift can work to facilitate the client’s explicit expression of his refusal to engage in chair work *in the moment* but, importantly, preserves the alliance (but [I mean I] I’m I’m open to anything) suggesting that the momentum in therapy, which was at risk, is not interrupted.

#### Direct Refusals

Clients may also directly refuse a therapist’s proposal with an unadorned “no”, as illustrated in Extract 10.


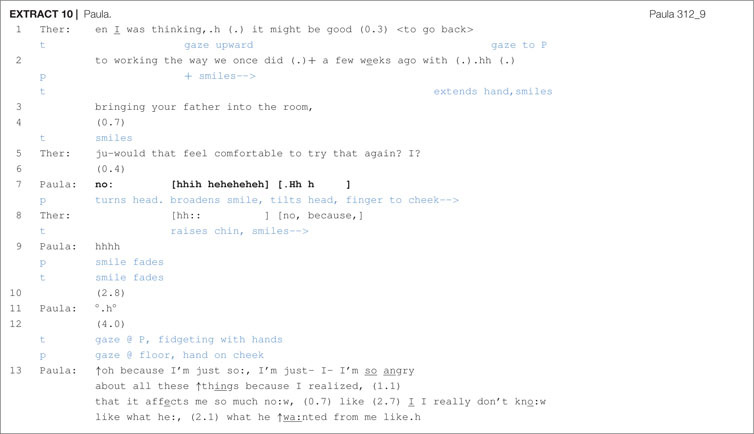


Following the therapist’s proposal to do chair work, lines 1–3, a silence ensues, which may be rejection implicative. The therapist smiles in line 4, which may be working to gain an affiliative return smile from the client (Bänninger-Huber, 1992), and then she produces a subsequent version of the proposal (Davidson, 1984), one that, because it more directly seeks confirmation, is more highly response relevant (Stivers and Rossano, 2010). Following a brief pause, Paula refuses (“no:”) and then quickly laughs while smiling. The therapist, in line 5, returns Paula’s smile, repeats the “no” and then seeks an account from Paula that explains or justifies her refusal. After silences and hesitation, Paula provides an account and the talk turns toward the father and the difficulty that Paula has in facing him in the chair at this moment in time. Unlike some of the other proposals shown previously, this proposal format lacked a clear orientation to contingency and to the client’s greater entitlement to give assent, but it also seemed not to display an elaborately articulated description or reformulation of the client’s trouble with the father. But by pursuing an account from the client, although the therapist was not able to get Paula to engage in chair work, the conversation was still able to shift toward a clearer focus on the father and some of the emotional difficulties surrounding her relationship with her father.

## Discussion

In-therapy tasks form a ubiquitous component in diverse forms of psychological treatments. They may range from free association (Freud, 1940) and chair work (Greenberg, 1979; Hill et al., 2012) to relaxation exercises and desensitization experiences (Russell and Lent, 1982). The rational for each of these tasks and the expected benefit varies, but in each case, the client is invited to engage in an activity that has the potential of inducing anxiety or stress, and therefore opposition. Refusing to engage in a task is doubly problematic for the progress of therapy. Clients may not only miss the opportunity to benefit from the proposed activity, but also refusal or avoidance may induce relational stress between the therapist and client, which may further precipitate an “alliance rupture”. If clients are opposing engagement with the task proposed by the therapist, they are evidently not in agreement about what is useful/desirable to do in the moment (Safran and Kraus, 2014).

There is an extensive literature examining the *reasons* clients might choose to contest therapists’ directives in different contexts (Beutler et al., 2002). We endeavored to compliment this literature by examining the sequential process and the discursive challenges associated with getting a “yes” to proposals of an in-therapy activity. From this perspective, we hoped to take some first steps to discover how certain conversational resources (within a specific sequence type) used by therapists can facilitate client engagement and overcome obstacles of mis-alignment. We began our investigation by first examining extant research on discursive practices on directives in general (e.g., Couper-Kuhlen, 2014; Landmark et al., 2015; Stevanovic and Svennevig, 2015; Kendrick and Drew, 2016). This literature alerted us to the importance of the deontic stance of the person wishing to direct another and the implicit and explicit position of power and authority of the proposer in relation to one who is asked to do something. From our previous work on relational negotiations we also knew that the epistemic claims/position of the prior interaction (getting agreement on the problem that requires task-based work) are likely to play a role in negotiating participation (Muntigl et al., 2017). Thus, it would seem that a lack of intersubjective alignment on the client’s emotional troubles – negotiated in phase 1 of chair work entry – might lead to hesitation and rejection, and also requires that the therapist do more accounting and elaborating.

To highlight and focus on the *process* of negotiation in therapy and how therapists deal with opposition or deferral, we chose to restrict our data to one specific task and context: Negotiating chair work in Emotion-focus therapy provided to clients diagnosed with depression. Our hope was that this narrow focus would permit us to more clearly identify the discursive practices that therapists successfully use to resolve incrementally more challenging levels of opposition to a proposed activity. From our corpus of proposal sequences, it was found that in the majority of cases acceptance was not immediate and that clients displayed some form of dissent via delays in responding or by more explicit refusals. Similar to the findings on directives in general (non-institutional) contexts, we found that successful negotiations involved therapists hoping to recruit clients to do chair work taking a flexible and appropriately responsive deontic stance. In smooth entries, the therapists’ proposals were developed using tentative, contingent forms such as “like to try”, “would you be willing to”, “wanna?”, “at least try”. Therapists also realized a distant non-authoritative position to the topic identified just prior to chair work using deictic forms such as “that” to refer to the topic. This served to downgrade the therapists’ epistemic stance and signaled deferment to the client’s authority and agency to formulate the content of chair work. Enfield and Sidnell (2017) have argued that the locus of agency is not in the individual, but rather in the social unit. Thus, as the goal of achieving alignment on a task is shared by the therapist/client dyad, the interplay of resources drawn on from both participants work to jointly accomplish future action/behavior. Proposals implicate that the activity to be done is collaborative, needing both participants to control how it will unfold. Contingent formulated proposals further orient to this shared and distributed agency by allocating more responsibility to the client, to confirm what is to happen next.

We provided examples of how silence, delay, or shifting of gaze can indicate opportunities for the therapist to engage with the clients’ subtly expressed opposition/reluctance. For example, in Extracts 7 and 8 the therapist responded to these minimal clues and sought confirmation and, thus, created an opportunity for the clients to either topicalize their concerns or objections or shift their position toward engagement. We noted the use of “or” as a way of offering/prompting the client to formulate or re-define the task. Therapists facing some level of reluctance also engaged in “accounting” or an elaboration and extension of the rationale for engagement in the task without directly re-iterating the request. These observations point to the utility of CA work in showing, for example, how speakers are not only constantly monitoring each other’s states of knowledge (or even their willingness to participate in a future activity), but are adjusting their contributions in response to these epistemic (and deontic) shifts (Goodwin, 2018). Importantly, in successful negotiations resulting in eventual engagement, therapists were sensitive to prosodic, non-verbal, as well as verbal indications of opposition or hesitation by the client. Thus, the therapists’ actions are not static, but often were modified, sometimes even mid-turn. What is shown in these exchanges is how therapists responsively shift their deontic positioning during moments in which acceptance is not forthcoming. This kind of moment-to-moment sensitivity closely parallels the positive therapist attribute of “Appropriate Responsiveness” discussed by Stiles and Horvath (2017).

## Limitations

Our sample of proposal/response sequences was relatively limited in size. Although we have been able to identify a variety of ways in which clients (implicitly) contest therapist proposals and therapists manage such reluctance/opposition, there are likely other resources and actions that could be doing this kind of interactional work. We did not attempt to canvass the variety of in-therapy tasks, nor did our data cover diverse treatments or a variety of psychological problems. The goal of this research was to examine practices to overcome/negotiate opposition to a challenging in-therapy task in a somewhat typical context. We anticipate that different tasks in different treatments will have some unique features not evident in our examples. However, the focus of this initial investigation was on aspects of negotiating dissent that we felt are likely shared with a range of challenging in-therapy activities in other contexts.

Working with active and passive reluctance/opposition has an enormous heritage in the literature, both theoretical and empirical. However, relatively little systematic research is available that focuses on how this dynamic is managed successfully as an interactive social achievement. The research we present offers an initial foray in identifying the conversational resources that are sequentially developed, both in clients taking a reluctant position and the ways in which such impasses may be resolved in therapy. A better understanding of how delayed engagement or refusals to perform in-therapy tasks are managed has a potential of making a practical contribution to therapist training and development.

## Conclusion/Recommendations

Our sample of directives to engage in chair work essentially captured “cooperative” negotiations. Even when engagement in chair work was refused, therapists and clients were able to manage reluctance to engage, repair the drift in task consensus, and maintain a collaborative momentum in therapy. An essential clinical practice in successfully managing reluctance to do chair work seems to be the development of a clear and shared understanding of what the conflict is *before* proposing the directive to engage. Such clarity generates an opportunity to use the deictic “that” as a reference point in the proposal and clarifies the potential benefits of doing chair work, as well as the risks of engagement. Taking a tentative flexible deontic stance creates generous opportunities for the therapist to find a collaborative position wherein clients may assume an agentive role and makes the proposal work for them. Likewise, careful attention and respect to the clients’ epistemic authority, supporting their awareness and expertise of their own issues and capacities, creates a context where the therapist can advocate the activity while the client feels supported and entitled to make choices.

Collaborative negotiations are typically responsive and incremental. Therapists approach the proposal to do chair work in an open ended, flexible way by developing an “or” position, seeking ongoing confirmation as the negotiation proceeds and being open to elaborate on and account for the rationale. This flexible/responsive approach is not only more likely to be productive in terms of engagement in the proposed work but will more likely preserve the alliance and therapy momentum if the client refuses to engage in the activity. Our CA study focused on recurring practices that generalized across cases. Future research on this topic using larger data sets (i.e., more sessions) might examine ‘typical’ practices occurring within a case and compare practices between cases and relate sequences of smooth vs. mediated entry (and opposition) with outcome and alliance measures.

## Data Availability Statement

All datasets presented in this study are included in the article/supplementary material.

## Ethics Statement

The study involving human participants was reviewed and approved by the Simon Fraser University Research Ethics Board. Written informed consent was obtained from the participants to participate in the York I study and for the publication of anonymized data.

## Author Contributions

PM wrote the analysis and produced the final edited manuscript. AH wrote Introduction and Conclusion. LC transcribed the chair work extracts, identified initial set of directive sequences, and provided copy editing. LA supplied the videorecorded sessions and helped to work on the content of the manuscript. All authors contributed to the article and approved the submitted version.

## Conflict of Interest

The authors declare that the research was conducted in the absence of any commercial or financial relationships that could be construed as a potential conflict of interest.

## Footnotes

1 For a fuller description of techniques involving the chair, see Perls (1973), Greenberg et al. (1993).

2 We are grateful to Drs. Greenberg and Watson for generously sharing the anonymized database of the York I study for our investigation.

3 One case was not transcribed due to time and financial limitations, leaving our total corpus to 7 (3 recovered, 4 unrecovered).

4 Especially when client agreement was not immediately forthcoming, therapists sometimes produced another (or multiple) proposal(s) to do chair work. Each proposal-response was taken as a separate sequence in this paper and, thus, our number of proposal sequences ended up being larger than the number of sessions analyzed (i.e., 18).

5 Although clients must assume ‘roles’ in chair work (e.g., ‘self’ vs. ‘significant other’; ‘criticized self’ vs. ‘critic’), it is not a form of role-play (cf. Stokoe, 2014); that is, unlike role-play, which is about simulating ‘real life’ events, often used as a basis for training, chair work aims at re-creating the catharsis of the person’s *real* past or “unfinished business”.

6 For practices of making descriptions more granular in psychotherapy/storytelling activities, see Muntigl et al. (2014).
